# Dementia care initiative in primary practice – study protocol of a cluster randomized trial on dementia management in a general practice setting

**DOI:** 10.1186/1472-6963-9-91

**Published:** 2009-06-06

**Authors:** Rolf Holle, Elmar Gräßel, Stefan Ruckdäschel, Sonja Wunder, Hilmar Mehlig, Peter Marx, Olaf Pirk, Martin Butzlaff, Simone Kunz, Jörg Lauterberg

**Affiliations:** 1 Institute of Health Economics and Health Care Management, Helmholtz Zentrum, München, Germany; 2 German Research Center for Environmental Health, Neuherberg, Germany; 3 Department of Psychiatry and Psychotherapy, Medical Psychology and Medical Sociology, University Hospital Erlangen, Erlangen, Germany; 4 HealthEcon AG, Basel, Switzerland; 5 AOK Bavaria, Nürnberg, Germany; 6 Eisai GmbH, Frankfurt, Germany; 7 Pfizer Germany GmbH, Berlin, Germany; 8 Health Economics and Outcomes Research, IMS Health GmbH & Co. OHG, Nürnberg, Germany; 9 Competence Center for General Practice and Outpatients' Health Care, Witten/Herdecke University, Witten, Germany; 10 Federal Association of the AOK, Berlin, Germany, and Institute for Patient Safety, University of Bonn, Bonn, Germany

## Abstract

**Background:**

Current guidelines for dementia care recommend the combination of drug therapy with non-pharmaceutical measures like counselling and social support. However, the scientific evidence concerning non-pharmaceutical interventions for dementia patients and their informal caregivers remains inconclusive. Targets of modern comprehensive dementia care are to enable patients to live at home as long and as independent as possible and to reduce the burden of caregivers. The objective of the study is to compare a complex intervention including caregiver support groups and counselling against usual care in terms of time to nursing home placement. In this paper the study protocol is described.

**Methods/Design:**

The IDA (Initiative Demenzversorgung in der Allgemeinmedizin) project is designed as a three armed cluster-randomized trial where dementia patients and their informal caregivers are recruited by general practitioners. Patients in the study region of Middle Franconia, Germany, are included if they have mild or moderate dementia, are at least 65 years old, and are members of the German AOK (Allgemeine Ortskrankenkasse) sickness fund. In the control group patients receive regular treatment, whereas in the two intervention groups general practitioners participate in a training course in evidence based dementia treatment, recommend support groups and offer counseling to the family caregivers either beginning at baseline or after the 1-year follow-up. The study recruitment and follow-up took place from July 2005 to January 2009. 303 general practitioners were randomized of which 129 recruited a total of 390 patients. Time to nursing home admission within the two year intervention and follow-up period is the primary endpoint. Secondary endpoints are cognitive status, activities of daily living, burden of care giving as well as healthcare costs. For an economic analysis from the societal perspective, data are collected from caregivers as well as by the use of routine data from statutory health insurance and long-term care insurance.

**Discussion:**

From a public health perspective, the IDA trial is expected to lead to evidence based results on the community effectiveness of non-pharmaceutical support measures for dementia patients and their caregivers in the primary care sector. For health policy makers it is necessary to make their decisions about financing new services based on strong knowledge about the acceptance of measures in the population and their cost-effectiveness.

**Trial registration:**

ISRCTN68329593

## Background

Dementia care is one of the most important challenges for health care systems in the greying societies of – for example – the US or Europe. Based on epidemiological prognoses they will have to cope with doubled or tripled figures of dementia patients until 2050 [1-3]. In Germany, direct medical costs for patients with dementia totalled 6.1 billion Euros in 2004 which made up 2.7% of total direct medical cost. Institutional care for patients with dementia accounted for 3.9 billion Euros [4]. Hallauer and colleagues calculated that the average total costs per patient including informal care sum up to approximately 44,000 Euros per year in Germany, of which the monetary equivalent of informal care giving time is the largest share [5]. From a societal and public health point of view, there is need to implement evidence based standards and services of medical and social care for patients and their caregivers [3].

Looking at current clinical dementia guidelines, there seems to be sufficient evidence for the efficacy of some drugs (e.g. cholinesterase inhibitors) in symptomatic therapy aiming at the delay of the disease progress. However, the scientific evidence concerning non-pharmaceutical interventions for dementia patients and their informal caregivers remains inconclusive [6-15]. Reasons can be found in the heterogeneity of intervention types, populations, settings, study designs, outcomes and – in addition – in widespread methodological quality problems, e.g. small sample sizes, observation periods too short to see clinically relevant intervention effects, and further shortcomings of scientific evaluation [6,7]. In a recently published meta analysis, an overall significant positive effect of non-pharmacological interventions in delaying institutionalization was reported, but there was significant heterogeneity between the thirteen studies [16]. While the cost-effectiveness of anti-dementive drugs is frequently analyzed, only few economic evaluations exist for non-medical interventions aiming to postpone institutional care [17-23].

Because in Germany general practitioners (GPs) play the key role in health care of the elderly, they automatically come into focus when introducing new services in dementia care. In most cases GPs are primarily faced with essential tasks like detecting and diagnosing dementia, information of patient and family members, drug therapy, and linking to further counselling and supportive measures. Especially in the care of non-institutionalized dementia patients, the GPs' influence on the help seeking behaviour of patients and caregivers can be decisive for families' willingness to accept external support and expert counselling. A systematic interdisciplinary collaboration between GPs, medical specialists and professionals in the field of social and psychological dementia care will be a key issue in the future [24].

In current practice the context of dementia care is difficult and study results [25-31] hint on the existence of a bundle of factors related to patient, caregiver, and physician which are building a barrier for optimizing dementia care.

On the societal level, dementia is a stigmatized disease surrounded by influences of ageism. Patients and caregivers often deny dementia symptoms, experience difficulties in accepting the diagnosis, and react to the disease with social withdrawal and depression. They have a lack of disease specific knowledge and of information on regional support and counselling services. This lack of knowledge is often shared by their GPs [32] who have a tendency of tabooing cognitive impairments and have difficulties in telling bad news – especially disclosing a diagnosis of dementia [33-36]. Furthermore, a skeptical or negative attitude towards the potential benefits of dementia drug therapies [37] and the unclear evidence concerning non-pharmaceutical treatment and support options as well as unclear referral ways to specialist psychiatric services (e.g. memory clinics) may prevent the optimal care for dementia patients.

All these above mentioned factors in sum lead to delayed and insufficient care with regard to diagnostics, treatment, counselling and social support for many dementia patients [3,26]. Therefore, a potentially successful intervention program in primary care has to address several of these obstacles to optimize dementia care. Focussing on single factors like education and training programs for doctors or distribution of dementia guidelines to close GPs' knowledge gaps probably is not enough [38-40].

The IDA study aims to combine several treatment options including training of GPs concerning diagnosis and treatment of dementia, out-patient specialist support for GPs, the offer of support programs and counseling for informal caregivers of patients with dementia.

The main research questions of the IDA project, from the perspectives of health care and health economics, are: (1) Is a complex intervention for community dwelling dementia patients, their caregivers and doctors more effective than usual care with respect to postponement of nursing home placement? (2) Does the intervention have an effect on disease progression and on caregiver burden? (3) Is the intervention cost-effective compared to usual care when assessed from the societal perspective? The objective of this paper is to describe the study protocol of the cluster-randomized IDA study. Recruitment and follow-up of all study patients have ended and the analysis phase has started by spring 2009. The detailed description of the study background and protocol is published as a reference for forthcoming papers describing the study results.

## Methods/Design

### Study Design and Setting

**Figure 1:**
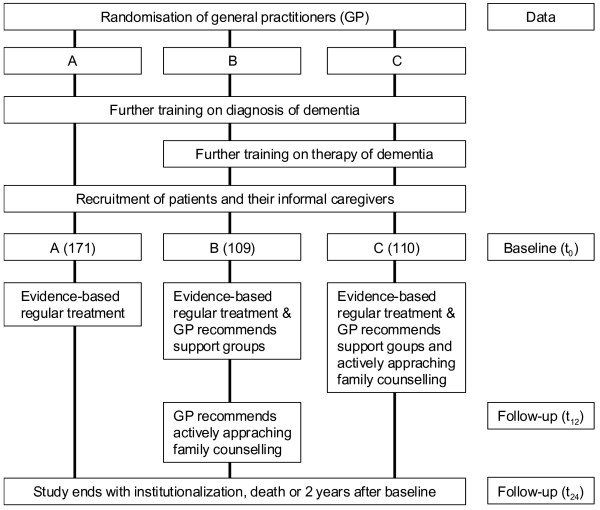
**Study design**.

The IDA study is designed as a three-armed cluster-randomized trial. The main research hypothesis is that for dementia patients still living at home a complex intervention consisting of an initial training of GPs in evidence based diagnosis and treatment, of the provision of caregiver support groups, and of actively approaching family counselling, can prolong time to nursing home placement in comparison to usual care. In comparison to usual care (study arm A), one intervention group receives additional training of GPs in medication and non-medication based therapies for dementia, and caregivers are offered to participate in caregiver support groups (study arm B), whereas in the second intervention group actively approaching family counselling is additionally implemented (study arm C). The duration of the intervention phase is two years. However, a change of study design became necessary due to the unexpected slow recruitment of patients (18 months instead of 6 months) and the very low participation rates of caregivers in the caregiver support groups. The main focus of the study was shifted towards the comparison of groups A and C. In group B, the actively approaching family counselling was implemented after the 1-year follow-up. Thereby, patients in arm B can serve as an intermediate intervention group in the first year and as an additional full intervention group in the second year. The new study design is shown in Figure 1.

The study region Middle Franconia is a mixed urban rural area around Nuremberg in Bavaria, Germany.

### Recruitment of GPs and Patients

All GPs in the study region in Middle Franconia (about 1200) were contacted with an information leaflet and invited to participate in the project. GPs willing to participate had to sign a contract with the AOK sickness fund. The training sessions (one for each GP) took place with groups of 10 to 30 GPs.

After the training, the GP identified all dementia patients in his or her practice who fulfilled the following selection criteria. Patients were included if they had mild to moderate dementia as defined by an MMSE (Mini Mental State Examination) between 10 and 24 [41], if they were at least 65 years old and a member of the AOK health insurance, and if they still lived at home and had an informal caregiver who was willing to participate in the study. The MMSE is a widely used test of cognitive function. Patients with a MMSE score below 10 were excluded because they were considered to be unable to give informed consent. In addition, we expected that in these patients nursing home placement could be necessary before treatment could have any effect. Patients with a MMSE score above 24 were excluded to gain a certain level of diagnostic accuracy and reduce the rate of falsely included patients with mild cognitive impairment (MCI), because a differential diagnosis is often difficult for GPs. Patients were excluded if they had terminal illness or if admission to nursing home was already planned. Patients not able or not willing to give informed consent were also excluded. Patients could be newly diagnosed or were already known as dementia patients.

Signed informed consent of study participants and their informal caregivers was required before patients could be entered into the study. The study protocol as well as the amendment necessitated by the change in study design has been approved by the Ethics Committee at the Bavarian Chamber of Physicians. (Date of approval: 30/05/2005, Reference number: 05029). Furthermore, the study is conducted in accordance to German privacy law and in compliance with the Helsinki Declaration. Recruitment of patients started in June 2005 and ended in December 2006.

### Randomization

The IDA project is a cluster-randomized study where the general practices are the clusters and thus the units of randomization. If two or three GPs from the same group practice wanted to participate in the study and attended the training course, they were randomized into the same study arm. The randomization was done by the statistics and data centre, usually one day before each training course when the full list of registered participants was available. We applied the randomization method of permuted blocks within strata, where stratification was done with respect to study region and type of practice (single vs. group). The allocation ratio was 1:1:1. The GPs were informed about their assigned study arm during the training course.

### Interventions

The study arm A serves as a control group where patients receive usual care. However, since GPs served as mediators of the intervention in IDA, all participating GPs received at least basic information concerning dementia diagnosis and about study procedures.

#### Training GPs

The obligatory training course for all participating GPs consisted of a dementia-specific part and a part concerning the study.

The dementia-specific part of the training consisted of dementia diagnostics including execution of the Mini-Mental Status Examination (MMSE) with a video demonstration and a section about patient and caregiver information (120 minutes). Afterwards, the GPs were informed to which study arm they were randomized. GPs of study arm A were separated from B and C and received study-specific information. Group A physicians were trained in early detection and diagnosis of dementia only. Their knowledge of non-medical treatment options and drugs was not part of the initial training. As a comparator this level of knowledge could serve as a proxy for the general status quo. The diagnostic training contained basic information about dementia (epidemiology, pathophysiology, early symptoms of dementia), anamnesis and physical examination, laboratory diagnostics, and psychometric tests. The additional training for group B and C physicians on treatment of dementia (140 minutes) consisted of information about interfaces in the German health care system, non-medication based treatment, information and counselling of caregivers, medical treatment options, therapy of non-cognitive disorders and specific problems. The dementia guideline for GPs, published by the Witten-Herdecke University  and the therapy recommendations of the Pharmaceutical Commission of the German Medical Association were used as the basis of the training. The therapeutic and diagnostic part of the training was given by five neurologists or psychiatrists with proven gerontopsychiatric expertise who are active in out-patient care in the study region.

All doctors received a folder containing written information specific to groups A, B, or C of the study including the slides of the training, the current dementia guideline and the baseline documentation forms for 10 patients.

#### Out-patient specialist support for GPs

The medical experts involved in the training offered out-patient consultant support to the GPs in groups B and C. In the case of acute deterioration or problems with therapy, appointments were available for dementia patients at short notice. Additionally a weekend telephone consultant on-call service was set up for the GPs.

#### Recommending family caregiver support groups

In groups B and C, the GPs suggested that caregivers should attend a family caregiver support group. The caregiver received an information sheet from the GP with contact details of 17 family caregiver support groups within the study region, all of which had been set up independently from the IDA project. The groups were required to have three qualifying characteristics: (1) professional supervision, (2) hold at least ten formal meetings per year, and (3) contain a psycho-educational element to improve the competencies of the family caregivers. After the consultation, the GP sent a fax to the chosen family caregiver support group, so that it would be possible to verify later which relatives attended a support group following the GP's offer. The family caregiver alone decided whether the visit actually took place or not.

#### "Counsellors contact caregivers" (CCC)

The family caregivers in Group C (and in group B after one year as well), were offered a special type of family caregiver counselling which would be recommended to them by their GP. After receiving the registration fax from the GP, the counsellor (now referred to as the "IDA counsellor") contacted the family caregiver by phone and tried to establish personal contact in form of a home visit, if possible. The IDA counsellor used elements of case and care management in educating and supporting the family caregivers psychologically and socially, to improve their competences in all facets of care. The aim was to support the family caregiver to the extent that the dementia patient was able to remain in the domestic environment as long as possible. Guidelines were developed for choosing those supportive measures that were expected to have a positive effect in specific situations. The IDA counsellor was also available by phone on weekends.

Four IDA counsellors worked in the project. Their professional competence consisted of training and several years of experience in nursing. They were expected to be able to empathise with the family caregivers and they received regular training in subjects important for the counselling process. Because of the large study area, the care managers were assigned to the patients regionally.

### Measurements

The study outcome variables concern potential effects of the intervention on the patient and on the caregiver level. In addition, economic outcomes from a societal perspective are registered.

The primary endpoint of the study is the remaining time the patient lives at home. This is defined as time from study entry until either nursing home placement (for at least 8 weeks) or death. In an additional analysis, death will be treated as a censoring event.

Secondary endpoints are:

1. Cognitive functioning of the patients (MMSE)

2. Patients' ability to perform ADL and IADL measured by the Barthel-Index [42] and the IADL subscale of the Nurses' Observation Scale for Geriatric Patients NOSGER [43]

3. Subjective burden of informal caregivers measured by the Burden Scale for Family Caregivers (BSFC) [44]

4. Quality of life of the patients (EQ-5D) [45-47]

5. Time to death

6. Number and duration of hospital stay

7. Direct medical and non-medical costs (including informal care)

8. Use of formal care and support services

Table 1 gives an overview on baseline and follow-up measurements. The measurements in the IDA project occur at the following fixed and variable time points: baseline measurement at entry into study (t_0_), follow-ups at six months (only for patients in arm B and C if they receive an antidementive drug) (t_6_), at twelve months (t_12_) and after 24 months (t_24_). If the patient was admitted to nursing home, no further assessments took place but GPs and caregivers were asked about the reasons for the institutionalization.

**Table 1:** Baseline and follow-up measurements

Aim	Instrument	Applied to	t_0_	t_6_	t_12_	t_24_	t_h_
Anamnestic data	Baseline CRF	GP	X				

Quality of life of patient	EQ-5D	Patient, informal caregiver and GP	X		X	X	

Cognitive function patient	MMSE	GP	X	X	X	X	

	DEMTECT	GP	X				

Evaluation of antidementiva therapy	CRF	GP		X			

Global health status of the patient	CGI	GP	X	X	X	X	

Behavioural problems patient	NOSGER (Subtest)	Informal caregiver	X		X	X	

ADL patient	Barthel-Index	Informal caregiver	X		X	X	

IADL patient	NOSGER (Subtest)	Informal caregiver	X		X	X	

Caregiver burden	BSFC	Informal caregiver	X		X	X	

Resource use, situation of caregiver	Questionnaire	Informal caregiver	X		X	X	

Nursing home placement (reasons)	Questionnaire	Informal caregiver and GP					X

The baseline assessment (t_0_) consisted of the documentation by the GP including test results and a telephone interview with the primary caregiver. In accordance with a naturalistic study design, documentation was restricted to an essential set of variables. The computer-aided telephone interview with the informal caregiver was conducted by trained interviewers. Apart from the Barthel-Index, NOSGER, and BSFC items, the telephone interview contains detailed assessment of informal care time and the use of formal care by home care services. If the interviewees refused to respond by telephone, they were asked to answer a written version of the questionnaire.

### Economic data

The cost-effectiveness of the intervention will be calculated from a societal perspective. Table 2 shows the different data sources for the assessment of resource use and direct cost. All patients, as well as caregivers who are insured with the same sickness fund, were asked to give permission to get their health insurance data over a time period covering one year before and four years after study entry.

**Table 2:** Data sources for cost estimation

*Category*	*Source*
Outpatient care	Health insurance data

Prescribed drugs	Health insurance data

Inpatient care	Health insurance data

Rehabilitation	Health insurance data

Medical aids and non-physician services	Health insurance data and caregiver interview

Informal care	Caregiver interview

Other dementia-related costs not reimbursed by the health insurance including self-paid drugs	Caregiver interview

Formal care and support services	Health insurance data and caregiver

The informal care time of all involved caregivers is assessed within the telephone interview with the primary caregiver. We used an extended version of the specific questions on informal care time of the Resource Utilization in Dementia (RUD)-instrument [48,49]. Formal and informal care was assessed by days during the last four weeks and average number of hours on these days. Costs not reimbursed by the health insurance were additionally assessed.

Intervention costs will be estimated, these include costs for care managers (including labour costs and overhead costs) and for caregiver support groups. Study specific costs, which would not occur in routine application, will not be considered.

### Measures against bias

The study is unblinded with respect to the GPs, the patients, and their caregivers. However, the assessment by telephone interview after one and two years is done by students who are not informed to which study arm the patient belongs.

From the methodological point of view, the main problem with cluster randomized trials is the potential for selection bias [50]. Although the GPs are randomly assigned to the study arms, inclusion of patients and their caregivers may be biased by foreknowledge of the allocation. This can occur because doctors may differently approach patients depending on the study arm they belong to or because patients and caregivers may differently consent to participation.

To address the problem of selection bias, we asked the participating GPs to initially provide a list of all dementia patients in their practice whom they considered eligible for the study. These lists will allow comparison of the basic characteristics of patients participating vs. those not included in the study overall and between study arms.

### Analysis

The confirmatory analysis of the study concerns the comparison of the three study groups with respect to the primary endpoint. To account for multiple testing, we plan to apply a closed testing procedure, which consists of testing the global null hypothesis of no difference between all three groups, and, in case of rejection, three subsequent pairwise comparisons with a significance level of 5%. Since the primary endpoint is a censored failure time variable, survival models adapted for clustered data will be applied [51]. All analyses will primarily be done according to the intention-to-treat approach. Analyses of secondary endpoints will use generalized linear models accounting for cluster randomization. Selection bias will be tested and adjusted for on the basis of available baseline data. For most secondary endpoints, no measurements will be available if the patient has died or moved into a nursing home. These analyses will therefore be done on the basis of all available cases. Additional analyses will be performed by applying imputation methods to those cases where patients were admitted to nursing home. In case of severely skewed distribution of cost variables, we will check the robustness of results by applying transformations or by performing bootstrap analyses.

### Sample size

For the sample size calculation, we had to consider a multiple test procedure for a three-group comparison with respect to a censored outcome variable and with allowance for a cluster effect. The calculation was based on an error probability α = 0.05 and a statistical power 1-β = 0.90 for each of the pairwise comparisons. As a clinically relevant effect we considered a difference of 15% or more in the probability a patient is still living at home after two years. The AD2000 Collaborative Group reported that 25% of the dementia patients had to be moved to a nursing home after two years [52]. This rate was used as event rate for study arm B. As deaths are counted as event we assumed a 10% point higher rate in all study arms. Under the alternative hypothesis we estimated that 50% (arm A, usual care) vs. 65% (arm B) vs. 80% (arm C) would still live at home after two years. As a result, 227 patients per arm were needed for a study with individual randomization [53]. Allowing for a cluster effect (ICC = 0.05, correction factor = 1.2) yields 272 patients per group. With further allowance for drop-out (10%) we decided to set the recruitment target at 900 patients. The change in study design reported above was considered acceptable with respect to statistical power, since a power of 75% could still be achieved for the comparison of groups A and C with a sample size as small as 120 per group if a 20% difference in event rates was assumed.

### Economic evaluation

The primary economic evaluation will be a cost-effectiveness analysis from the societal perspective. The results will be presented as incremental cost per additional year living at home. We will use the bootstrap approach to calculate confidence intervals for incremental cost-effectiveness ratios. In a secondary cost-utility analysis, quality-adjusted life years (QALYs) will be calculated based on EQ-5D values.

## Discussion

In this paper we describe the study design of an innovative cluster-randomized trial evaluating the cost-effectiveness of a complex intervention for GPs and family caregivers of patients with dementia living at home. The IDA study aims to combine several treatment options including training of GPs concerning diagnosis and treatment of dementia, out-patient specialist support for GPs, and support programs and counseling for informal caregivers of patients with dementia. The counseling for caregivers is the main intervention of the IDA study.

Particular strengths of the study design are the relatively large sample size, the cluster randomization, the long follow-up of two years, and the availability of complete health insurance data for all patients over the whole study period. Since we planned this study as a so-called pragmatic (or naturalistic) trial, we tried to aim at maximum generalizability by recruiting a large number of GPs who cover a mixed urban and rural region typical for Germany. We motivated participating GPs to include all suitable patients in the study. Formal inclusion and exclusion criteria for patients, such as availability of caregiver, were necessary and do not pose a threat to external validity. Restriction of patients to those from the AOK sickness fund seemed necessary, since we wanted to obtain complete health insurance data. This is no severe threat to external validity, since the regional AOK sickness funds have the largest share of insured patients in Germany. In 2005, about one million people or 49.6% of the population aged over 65 of the statutory health insurance market in Bavaria was insured by the AOK.

The major problem with respect to selection bias is the exclusion of patients whose caregivers are not willing to participate, either because of fear of stigmatization or other reasons. Some GPs may be more willing and able to convince patients and caregivers to participate than others, and in a cluster randomized trial this may also be related to the treatment group. In order to investigate selection bias, we plan to compare groups with respect to baseline data. In addition, we asked all GPs to initially provide a list of patients they would approach. In a subsample of about 50% of all GPs we conducted interim telephone interviews to ask them about progress of recruitment and reasons for non-inclusion.

Since patients with dementia may not be able to give correct information, we collected all data via GPs or caregivers or from the sickness fund. The only exception is assessment of quality of life, which was also collected from patients.

We chose time to nursing home referral as the primary endpoint because the main concept was to help caregivers to care for their relatives suffering from dementia as long as possible in their living environment. In a recent meta-analysis only studies were selected that reported this endpoint [16]. We discussed in detail whether death before nursing home placement should be included in the primary endpoint or count as a censoring event. Other studies often do not explicitly mention how this is handled as for instance Spijker et al 2008 [16]. It was decided to include it because this would minimize any possibility of bias and we expected to be able to accept the additional random error because of the large sample size. According to data from a population based study, median survival time is five to seven years from time of diagnosis in patients with mild or moderate dementia [54]. The outcomes nursing home placement and death can be completely assessed by AOK data. Reasons for nursing home placement may be unrelated to progress of dementia, e.g. caused by other disease or by death of a caregiver. We try to document these reasons as given by the GP and the caregiver wherever possible.

Two year follow-up was considered to be necessary in order to register enough events within the duration of the study. In our sample size calculation we expected to observe 50% events within two years based on estimations from the AD2000 study [52].

There was no additional validation of the dementia diagnosis as we decided to choose a naturalistic design. However, all participating GPs had a training unit in standardized application of the MMSE.

Physicians prescribe the drug that they consider as the right one for their patient, reflecting their knowledge of the disease, the diagnosis and the characteristics of the patient. In contrast to study arm A, GPs randomized in study arm B and C were trained in treatment of anti-dementive drugs. The aim of the training was to improve the knowledge of the physicians so that the treatment follows the guideline for GPs (Witten-Herdecke University) and the therapy recommendations of the Pharmaceutical Commission of the German Medical Association. Therefore drug treatment followed evidence-based guidelines and drugs were prescribed according to their approval. Differential drug prescription may have an additional intervention effect which cannot accurately be distinguished from the effect of the non-medical interventions. However, the prescribing pattern of the GPs in the three study arms will be known from the health insurance data and will be considered in the analysis.

To prevent intervention effects depending on the family counsellor we implemented a guideline for the counsellors. However, the process of counselling is individual and depends on many different factors such as the caregiver situation, the patients' health status, the living arrangement etc. Therefore, we cannot guarantee a unique counselling effect for all counsellors.

We experienced some specific unforeseen problems in the implementation of the study design that we were able to solve in a pragmatic way. During recruitment the question arose how to deal with caregivers who care for two people meeting the inclusion criteria. We decided that both patients could take part in the study. Questions within the telephone interview relating to the informal caregiver were only assessed once.

It was planned to recruit the primary informal caregiver of the patient that is the person who carries out the largest part of informal care. However, sometimes it was not the primary informal caregiver who signed the written consent. In those cases we refrained from contacting the primary caregiver and involved the non-primary caregiver. It might be possible, that their statements on resource use are less valid as they are not as strongly involved in the caring process as the primary informal caregiver.

Some informal caregivers preferred to answer the print version of the questionnaire. If the written questionnaires had values missing, we refrained from contacting these subjects again. Answers of the written questionnaire might differ from those of the telephone interview as caregivers have not the possibility to pose clarification questions.

The resource use of informal care is measured since it plays a substantial role in caring for patients with dementia living at home [55-59]. Omission of costs of informal care would bias cost-effectiveness against formal care in favor of informal care which seems to be free of charge [56]. Measuring the economic burden of informal care is not easy for several reasons. We decided to assess and value the time the informal caregivers spent on caring for the patient. However, sometimes it is not easy to distinguish between normal housework and informal care [60]. Furthermore, different methods are available for valuing that time [61].

However, this kind of valuation does not consider possible positive effects of care giving for the informal caregiver [62,63]. However, up to now there is no consent on how informal care should be incorporated in economic evaluation.

Due to the extension of the recruitment and intervention time the counselling will be offered to some caregivers for more than 2 years. This will allow us to analyze effects of the intervention on nursing home admission beyond the planned 2-year follow-up. Furthermore, patient and caregivers agreed to provide their health care insurance data for a period of four years after study entry which allows additional long-term analysis concerning the primary end point as well as cost from a health care perspective.

From a public health perspective the IDA trial should lead to evidence based results with regard to the community effectiveness of non-medical support measures for dementia patients and their caregivers in the primary care sector. For health policy makers it is necessary to make their decisions about financing new services based on strong knowledge about the acceptance of measures in the population and their cost-effectiveness.

## Competing interests

The sponsors have commissioned two academic research institutions with the scientific evaluation of the IDA project by giving unconditional research funds. A contract between the sponsors and academic researchers ensures that the latter have the full scientific responsibility and have the right to publish the results. Members of the sponsoring organizations closely cooperate in design and conduct of the project, but only the academic researchers have full access to all of the data in this study and take complete responsibility for the integrity of the data and the accuracy of the data analysis.

RH, EG, and SK are independent scientists who have received funding for this study as described above. MB and OP have received funding for cooperation in the design of the study. JL, SR, SW, PM, and HM are or have been employees of the sponsoring institutions.

## Authors' contributions

RH is principal statistical investigator, participated in the design of the study, and drafted the manuscript. EG is principal clinical investigator, participated in the design of the study, and helped to draft the manuscript. JL, HM, SR and PM conceived of the study, and participated in its design and coordination and helped to draft the manuscript. SK participated in the study design and coordination and performs the economic evaluation. SW is responsible for the local coordination of the study. OP and MB both helped to conceive of the study and contributed to the study design. All authors contributed to the manuscript and have read and approved the final version of the manuscript.

## Pre-publication history

The pre-publication history for this paper can be accessed here:


